# Effectiveness of a one-year smoking cessation intervention for people with severe mental illness: results of the KISMET cluster-randomized controlled trial

**DOI:** 10.1017/S0033291726104516

**Published:** 2026-05-15

**Authors:** Müge Küçükaksu, Trynke Hoekstra, Jentien M. Vermeulen, Lola Jansen, Sanne Helmig, Berno van Meijel, Marcel C. Adriaanse

**Affiliations:** 1 Department of Health Sciences, Vrije Universiteit Amsterdam, Amsterdam, Netherlands; 2 Department of Psychiatry, Amsterdam UMC - Locatie AMC: Amsterdam UMC Locatie AMC, Netherlands; 3 Department of Psychiatry, Amsterdam UMC - VUMC location: Amsterdam UMC Locatie VUmc, Netherlands

**Keywords:** randomised controlled trial, severe mental illness, outpatient mental healthcare, smoking cessation, tobacco addiction, cognitive-behavioural therapy, peer support, nicotine replacement therapy, pharmacotherapy

## Abstract

**Background:**

This study evaluated the effectiveness of a one-year smoking cessation intervention for people with severe mental illness compared with treatment as usual (TAU) in outpatient mental healthcare.

**Methods:**

The KISMET study is a pragmatic cluster-randomized controlled trial conducted in 21 outpatient mental healthcare teams in the Netherlands. Eleven teams delivered the KISMET intervention comprising cognitive-behavioral and peer support, combined with optional pharmacological reatment. Ten teams participated in the TAU condition. We collected data between October 2022 and July 2024. The primary outcome was self-reported smoking cessation at 12 months, verified through exhaled carbon monoxide levels below 10 parts per million. Secondary outcomes included depression and anxiety (HADS), severity of psychotic symptoms (PANSS-6), quality of life (SF-12), disease self-management (PAM-13), lipid profile, blood pressure, body mass index, glucose level, and physical fitness. Crude and adjusted linear and multivariable logistic regression and mixed model analyses were performed.

**Results:**

Eighty-nine participants were included in the KISMET intervention and 44 in TAU. Smoking cessation rates were significantly higher in the KISMET group at 3 months (OR 12.1, 95% CI 1.4 to 103.7) and at 12 months (OR 4.2, 95% CI 1.0 to 17.2) but not at 6 months (OR 1.9, 95% CI 0.5 to 6.9). No significant differences between groups were found for secondary outcomes. Dropout rates were 58% in the intervention and 32% in the TAU group.

**Conclusions:**

The KISMET intervention shows potential without signs of physical or psychopathological complications. However, results must be interpreted with the high dropout rates in mind.

## Introduction

People with severe mental illness (SMI) are approximately twice as likely to smoke than people in the general population (Lasser et al., 2000; Szatkowski & McNeill, 2015). Smoking significantly contributes to serious somatic diseases, a shortened lifespan by up to 25 years, as well as the exacerbation of psychiatric symptoms such as anxiety and depression (Prochaska, Das, & Young-Wolff, 2017; Walker, McGee, & Druss, 2015; World Health Organization, 2018). In the Netherlands, flexible assertive community treatment (FACT) teams provide outpatient care to people with SMI (Delespaul, 2013). Studies show that FACT professionals are motivated and feel capable to provide smoking cessation support to their patients (Blankers et al., 2016; Sheals, Tombor, McNeill, & Shahab, 2016). Many individuals with SMI want to quit smoking and want support from their mental healthcare provider to succeed in this (Brown et al., 2015; Chen et al., 2017; Malone, Harrison, & Daker-White, 2018). However, opportunities for systematic delivery of smoking cessation support are currently underused by MHPs (Blankers et al., 2016; Huddlestone et al., 2022).

There are clinical trials that have shown positive effects of combined pharmacological treatment and cognitive-behavioral support for smoking cessation in people with SMI (Daumit et al., 2023; Gilbody et al., 2019, 2021; Peckham et al., 2017; Spanakis et al., 2022). These studies and reviews demonstrated that bespoke face-to-face interventions for people with SMI are more effective in reducing smoking prevalence compared to usual care. However, there is limited evidence that additional peer support can make a positive contribution to smoking cessation. Peer support has been shown to be helpful for people with psychotic disorders in general (Castelein et al., 2008; Castelein, Mulder, & Bruggeman, 2008). The aim is to provide peer-to-peer interactions focusing on recovery and empowerment while creating a bond through similar life experiences (Boevink, 2017). The group may be guided by a mental healthcare professional with minimal involvement or an expert-by-experience who is trained in offering guidance based on their own experiences. Typically, participants autonomously choose the topic they want to discuss or activity themselves. A positive effect of peer support has also been shown for smoking cessation in particular (Dickerson et al., 2016; McKay & Dickerson, 2012; Yuan et al., 2023), but has not been investigated as a part of an integrative smoking cessation program.

Previously, we designed a smoking cessation program employing the clinical experience and knowledge of experts: the KISMET intervention (smoKing cessation Intervention for people with Severe Mental illnEss Trial) (Küçükaksu et al., 2022). Whether a comprehensive smoking cessation intervention is effective for people who receive treatment in FACT teams in the Netherlands has not been established yet. To the best of our knowledge, this is the first intervention for people with SMI combining pharmacological treatment, cognitive-behavioral support, and peer support for smoking cessation. We aimed to evaluate the effectiveness of the one-year KISMET intervention in Dutch mental healthcare compared to treatment as usual. We also conducted a process evaluation embedded in this RCT entailing interviews with 10 mental healthcare professionals and 16 patients. The outcomes of this qualitative study (reported elsewhere) open the ‘black box’ of our RCT and allowed us to investigate experiences of patients and professionals with the intervention more deeply (Küçükaksu, Jansen, Hoekstra, Helmig, Adriaanse, & van Meijel, 2025a, 2025b).

## Methods

### Study design

We conducted a pragmatic cluster-randomized controlled trial (RCT) in 21 mental healthcare teams from 11 organizations across the Netherlands. Twenty FACT teams and one early-intervention team (EIT) participated, out of which 11 sites delivered the KISMET intervention, while 10 sites participated in the treatment as usual (TAU) condition. Data were collected between October 2022 and July 2024 at four time points throughout the one-year study period: at baseline (prior to the start of the intervention), and followed up at 3, 6, and 12 months. The study protocol has previously been published (Küçükaksu et al., 2023).

### Setting and participants

The KISMET intervention was implemented within FACT teams and one EIT team. The decision to include one EIT team, which was recruited through snowball sampling, was made based on the team’s high motivation to participate and their outreaching care model, similar to that of FACT teams. FACT and EIT teams are multidisciplinary teams that provide long-term mental healthcare to people with SMI, usually delivered within patients’ home environment. Each team consists of a psychiatrist, psychologists, nurses, clinical nurse specialists (CNS), social workers, and experts-by-experience, delivering care within a certain geographical area to a caseload of averagely 200 patients (van Veldhuizen et al., 2015). The intensity of care and treatment can be tailored to the needs of the individual patient, with the possibility of providing intensive outreach care.

Patients were eligible to participate if they were 18 years or older, capable to consent, smoked on a daily basis (>5 cigarettes per day), expressed a wish to quit or reduce smoking, and diagnosed with SMI by a qualified mental healthcare professional prior to recruitment, as defined by Delespaul (2013): presenting a non-transient psychiatric disorder, for which coordinated care from a care network is necessary and which is not in symptomatic remission; presenting severely impaired social and/or occupational functioning which is not in functional remission. Exclusion criteria were contraindications due to an acute somatic disease or psychiatric crisis assessed by a physician or psychiatrist, pregnancy or breastfeeding, or a primary diagnosis of substance use disorder.

### Recruitment

We approached 19 mental healthcare organizations, out of which 11 agreed to participate. In total, 85 FACT teams were approached within these 11 mental healthcare organizations and informed about the study by research staff. Managers of mental healthcare organizations were our first point of contact and introduced the study either in all FACT teams or in pre-selection based on suitability (e.g. sufficient staff available). Enthusiastic FACT teams could then indicate they are interested to participate and appoint one or two mental healthcare professionals who would be the point of contact. Subsequently, we would contact interested FACT teams and give more detailed information of what participation would entail. Eventually, 21 teams decided to participate.

Starting on 13 September 2022 patients were approached by their mental healthcare providers, either face-to-face or by phone. Flyers and posters advertising the study were displayed in mental healthcare facilities to inform patients about the study. Contact details of one researcher (MK) were included on these flyers and posters so patients could take the initiative to participate in the study. Patients were informed about the aim and procedures of the study, both orally and with an information letter. After a minimum of 7 days of time for consideration, patients were asked whether they wished to participate. Written consent to participate was taken between 17 October 2022 and 21 July 2023.

### Cluster-randomization

Cluster-randomization was performed on a FACT team level within mental healthcare organizations. Due to the group setting of the intervention and the training that MHPs received, randomization on a patient level might have led to contamination within teams (Torgerson, 2001). FACT teams were randomly assigned to either the KISMET intervention or TAU. Computerized randomization was conducted by a statistician not directly involved in the study, using a random number sequence. Blinding the researchers, participants or MHPs was not possible due to the nature of the intervention.

### KISMET intervention

Between October 2022 and May 2024, FACT teams delivered the KISMET intervention, consisting of 20 group sessions based on cognitive-behavioral therapy and motivational interviewing (MI), a peer support group and optional pharmacological treatment. Group sessions were led by a nurse/CNS and/or a psychologist. Peer support sessions were led by an expert-by-experience or MHPs who quit smoking in the past. Group and peer support sessions took place weekly in the first 3 months, then monthly. Group sessions incorporated psychoeducation about tobacco addiction and associated health risks, potential withdrawal symptoms, pharmacological treatment options and potential side effects, setting personal goals, identifying triggers and high-risk situations, a relapse prevention plan and behavioral activation. This approach can support smoking cessation by improving mood and motivation, distracting from cravings, or providing a reward for remaining abstinent. An overview of the sessions can be found in Supplementary Materials (S1). Pharmacotherapy was supervised by a CNS or psychiatrist and included nicotine replacement therapy (NRT) as first choice (nicotine patches and chewing gum), or as a second choice Cytisine, Bupropion or Nortriptyline. These medications are in accordance with national and international treatment standard guidelines for smoking cessation (NHS, 2019). The KISMET intervention was systematically developed in collaboration with relevant stakeholders, including an expert-by-experience with severe mental illness and mental healthcare professionals, and based on existing evidence. Expert consensus was established on the core components. Additionally, strategies to optimize practical implementation were formulated (Küçükaksu et al., 2022).

### Treatment as usual

Patients in FACT teams in the TAU condition had access to standard care for smoking cessation. This could include a referral to the general practitioner, prescription of smoking cessation medication and/or participation in a smoking cessation program upon own initiative.

### Training and instructional meetings

FACT teams assigned to the KISMET intervention, participated in a one-day training informing about the three intervention components: group sessions, pharmacological treatment, and peer support. The three training elements were delivered by a psychologist, a physician, and an expert-by-experience, all having extensive relevant expertise. FACT teams received all necessary materials, such as a carbon monoxide (CO) monitor, the KISMET handbook, medication guidelines, and workbooks for patients.

We set up in-person or online meetings with FACT teams assigned to the TAU condition to give MHPs instructions about the study procedures and data collection. FACT teams in the TAU condition conducted all of the study assessments. We decided to do the data collection in most FACT teams in the intervention condition to reduce their burden. To ensure standardized data collection, we designed detailed data collection protocols.

### Outcome measures

#### Primary outcome

*Smoking cessation.* The primary outcome was smoking cessation at 12 months (Hughes et al., 2003). Smoking cessation was determined through self-report (i.e. abstinent in the last 7 days) and verified by exhaled CO level lower than 10 parts per million (Benowitz et al., 2020).

### Secondary outcomes

#### Psychological outcomes

Secondary outcomes were depression and anxiety (Hospital Anxiety and Depression Scale, HADS) (Spinhoven et al., 1997), severity of psychotic symptoms assessed by MHPs (short version of the Positive and Negative Syndrome Scale, PANSS-6) (Østergaard et al., 2016), disease self-management (13-item Patient Activation Measure, PAM-13®) (Rademakers et al., 2012), and quality of life (12-item Short-Form Survey, SF-12) (Ware, Kosinski, & Keller, 1996).

#### Physiological outcomes

Physical health was operationalized through body mass index, lipid profile, fasting glucose level, blood pressure, and a 6-minute walking test for physical fitness (Balke, 1963). Baseline nicotine dependence (6-item Fagerström Test for Nicotine Dependence, FTND) (Heatherton, Kozlowski, Frecker, & Fagerstrom, 1991), average number of cigarettes smoked per day calculated through the 7-day point prevalence, and cannabis use were presented (WHO ASSIST Working Group, 2002).

In addition, at 12 months follow-up, we registered quit attempts made in the past year in both groups and any received smoking cessation support in the TAU condition. Serious adverse events (SAEs) and dropouts (including reasons) were followed up and registered continuously throughout the trial. Finally, patients’ attendance at group sessions was recorded by MHPs in the intervention group.

### Demographics

We collected data on age, gender, education, and relationship status. Primary mental health diagnosis (DSM-5 classification) and use of psychotropic medication were retrieved from patient records.

#### Statistical analysis

Main analyses followed the intention-to-treat principle. Descriptive information was presented for the KISMET intervention and TAU separately. Continuous variables were presented as means (±SD) and categorical variables as proportions (%). Additionally, we compared participants who completed the study with those who dropped out (Supplementary Materials S2–S4). For the intervention group, we compared smoking quit rates in relation to attendance at group sessions. Overall frequencies of serious adverse events were analyzed for both groups, including the relation to our study.

We analyzed differences between the KISMET intervention and TAU in the primary outcome (smoking status) between baseline, 3 months, 6 months, and 12 months follow-up. Due to convergence- and data sparsity issues, we used separate logistic regression models (crude and adjusted for gender and age) per time point. We re-ran these analyses treating participants who dropped out as currently smokers and present both results.

Furthermore, initial sensitivity analyses proposed in our protocol paper regarding participants randomized to the KISMET condition, but who did not receive the intervention with none or only baseline visits are redundant (De Boer, Waterlander, Kuijper, Steenhuis, & Twisk, 2015; Küçükaksu et al., 2023). We reported odds ratios, corresponding 95% confidence intervals, and *P* values (significance at *P* < .05).

Next, we analyzed differences between the KISMET intervention and TAU in the secondary outcomes between baseline, 6 months, and 12 months follow-up (for severity of psychotic symptoms, we additionally analyzed differences between baseline and 3 months follow-up). For these analyses, we followed the common analysis strategy described by Twisk to adequately account for differences between the two groups at baseline (De Boer et al., 2015). In these mixed models, time (categorical variable), allocation group, the interaction between allocation group, and time were included. A random intercept on the patient and on the FACT team level was added to consider the clustering of the data structure (Twisk, 2006). We ran crude models, which already included a correction for baseline differences (included as a time-independent variable) in the respective outcome, and adjusted models, which additionally included gender and age (Smith et al., 2016; Steinberg et al., 2006; Torchalla, Okoli, Hemsing, & Greaves, 2011). Finally, we ran ‘overall’ models, assessing the average difference between the KISMET and TAU group for each outcome over time. All analyses were conducted in SPSS 29.0 and Stata Corp SE 18.

## Results

### Patient flow and sample characteristics

**Figure 1.**
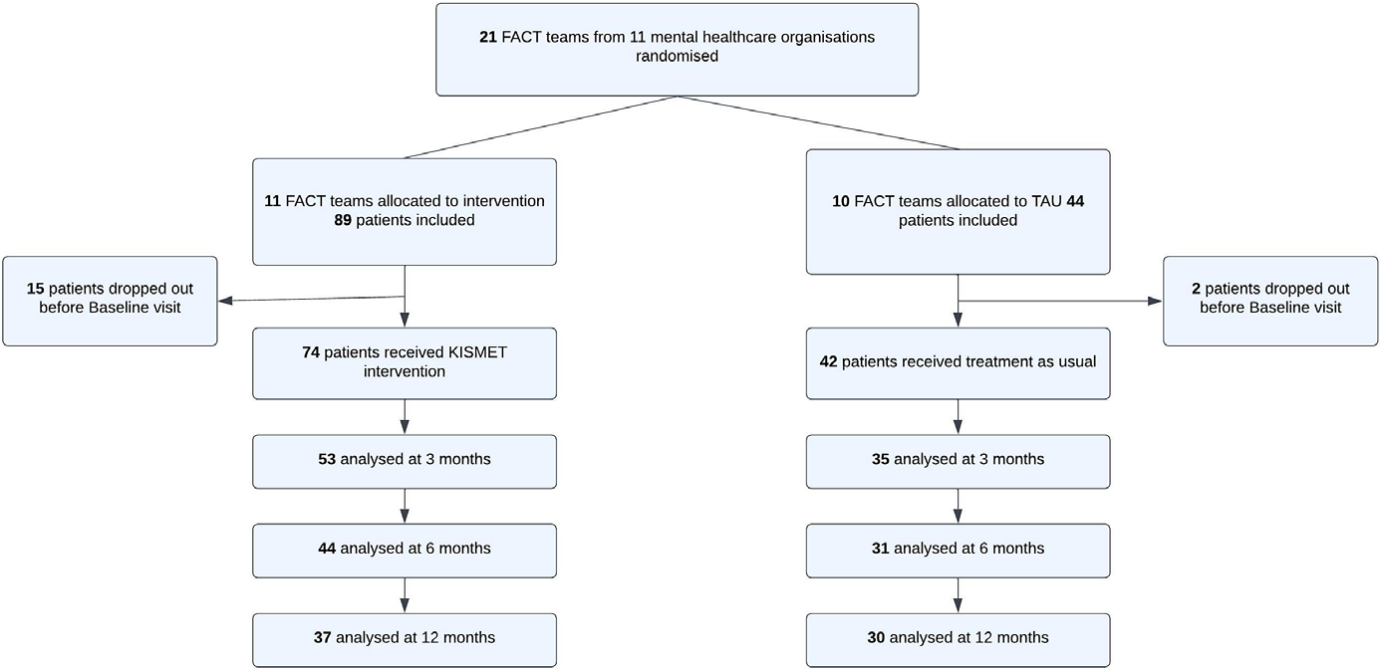
Flowchart of participants throughout the trial.

We recruited a total of 133 participants. The original aim was to recruit 318 participants. The study population included 89 participants from 11 KISMET intervention teams and 44 participants from 10 TAU teams at baseline. Figure 1 shows an overview of the number of initial inclusions and drop-outs throughout the one-year trial period.

Baseline characteristics are presented in Table 1. Mean age was 47.2 years (±SD 11.4) in the intervention group and 46.3 years (±SD 12.1) in the TAU group. There were slightly more women than men in the intervention group (52.7%), while more participants in the TAU group were men (64.3%). In both groups, most of the participants had a primary diagnosis of schizophrenia spectrum or other psychotic disorder, with 59.2% and 39% in the intervention and TAU group, respectively. The most frequently used antipsychotic medications were clozapine, olanzapine, and quetiapine. Baseline differences between participants who completed the study and participants who dropped out can be found in Supplementary Materials (S2–S4).

**Table 1.** Baseline characteristics of participants in the KISMET and TAU group

Characteristics	KISMET (N = 74)	TAU (*N* = 42)
Demographic characteristics		
Age (mean years, ±SD)	47.2 (11.4)	46.3 (12.1)
Gender, *N* (%)		
Woman	39 (52.7)	15 (35.7)
Man	35 (47.3)	27 (64.3)
In a relationship, *N* (%)	12 (16.2)	11 (26.2)
Single/divorced/widowed, *N* (%)	62 (83.8)	31 (73.8)
Education level, *N* (%)		
Low	21 (28.4)	11 (26.2)
Middle	38 (51.4)	24 (57.1)
High	15 (20.3)	7 (16.7)
Primary diagnosis (DSM–5)		
Primary diagnosis, *N* (%)		
Schizophrenia spectrum and other psychotic disorders	45 (59.2)	16 (39)
Bipolar disorder	8 (10.5)	1 (2.4)
Depressive or anxiety disorder	12 (15.8)	11 (26.8)
Personality disorder	5 (6.6)	7 (17.1)
Autism spectrum	2 (2.6)	2 (4.9)
Other psychiatric disorder	4 (5.3)	4 (9.8)
Physiological parameters & smoking		
Body mass index (kg/m^2^), mean (±SD)	28.9 (7.5)	27.9 (5.4)
Blood pressure (mm Hg), mean (±SD)		
Systolic	119 (18.1)	123 (14.0)
Diastolic	82 (9.4)	81 (11.8)
Cholesterol (mmol/l), mean (±SD)		
Total	5.2 (1.1)	4.8 (1.14)
HDL	1.2 (0.3)	1.3 (0.6)
LDL	3.2 (0.9)	3.0 (0.8)
Triglycerides	2.1 (1.1)	2.0 (1.5)
Fasting glucose (mmol/l), mean (±SD)	6.3 (2.2)	5.8 (2.1)
Average number of cigarettes per day, mean (±SD)	19 (10.7)	17 (9.4)
Years of smoking, mean (±SD)	26.4 (11.3)	27.3 (13.7)
Carbon monoxide level (ppm), mean (±SD)	22 (11.9)	25 (16.8)
E-cigarette/vape use, *N* (%)	4 (5.4)	8 (19)
Cannabis use last 12 months, *N* (%)	19 (26.8)	11 (27.5)
Psychopathological parameters		
FTND degree of nicotine dependence^a^, means (±SD)	5.9 (1.4)	5.6 (1.7)
HADS anxiety scale^b^, means (±SD)	9.1 (4.9)	8.2 (5.5)
HADS depression scale^b^, means (±SD)	7.6 (4.5)	7.2 (5.1)
PANSS–6 psychotic symptoms^c^, means (±SD)	13.2 (6.6)	10.8 (5.0)
Use of psychotropic medication, *N* (%)		
Use of antipsychotic medication	46 (51.7)	26 (59.1)
Olanzapine	11 (23.9)	5 (19.2)
Clozapine	10 (21.7)	5 (19.2)
Quetiapine	9 (19.6)	4 (15.4)
Other	16 (34.8)	12 (46.2)
Use of antidepressant	51 (57.3)	32 (72.7)

*Note*: FTND, Fagerström Test for Nicotine Dependence; DSM-5, Diagnostic and Statistical Manual of Mental Disorders Fifth Edition; HADS, Hospital Anxiety and Depression Scale; HDL, high-density lipoprotein; LDL, low-density lipoprotein; PANSS-6, Positive and Negative Syndrome Scale; SD, standard deviation.

a Scored on a 0–10 scale ranging from no nicotine dependence to very high level of nicotine dependence.

b Scored on a 0–21 scale, with higher scores indicating levels of anxiety or depression.

c Scored on a 6–42 scale, with higher scores indicating severity of psychotic symptoms (6 = absent; 42 = extreme).

### Primary outcome

#### Smoking cessation

After 3 months, 12 participants (22.6% of remaining participants) in the KISMET intervention had quit smoking, compared to one participant (2.9%) in the TAU group. At 6 months, 10 participants (22.7%) in the KISMET intervention had quit smoking, compared to 4 participants (12.9%) in the TAU group. After 12 months, 12 participants (32.4%) in the intervention group had quit smoking, and 3 participants (10%) in the TAU group (Supplementary Materials S5). Crude and adjusted odds ratios with corresponding 95% confidence intervals are presented in Table 2 and indicate statistically significant intervention effects at 3 and 12 months but not at 6 months follow-up. Analyses treating participants who dropped out as currently smoking show overall slightly attenuated, or similar, effect sizes (see Table 3).

**Table 2.** Crude and adjusted effects of the KISMET intervention on smoking cessation

	Crude model			Adjusted model^a^		
	Odds ratio^b^	95% CI	*P* value	Odds ratio	95% CI	*P* value
3 months	9.7	(1.2 to 78.2)	0.034	12.1	(1.4 to 103.7)	0.023
6 months	1.9	(0.5 to 6.8)	0.316	1.9	(0.5 to 6.9)	0.347
12 months	4.2	(1.1 to 16.5)	0.043	4.2	(1.0 to 17.2)	0.047

a Adjusted for gender, age.

b Odds ratio can be interpreted as the odds of having quit smoking at the respective timepoint for the KISMET intervention compared to TAU.

**Table 3.** Crude and adjusted effects of the KISMET intervention on smoking cessation, treating participants who dropped out as currently smoking

	Crude model			Adjusted model^a^		
	Odds ratio^b^	95% CI	*P* value	Odds ratio	95% CI	*P* value
3 months	7.9	(1.0 to 62.9)	0.052	8.6	(1.1 to 69.7)	0.043
6 months	2.0	(0.5 to 7.8)	0.311	2.1	(0.5 to 8.4)	0.282
12 months	3.8	(0.8 to 18.1)	0.089	4.1	(0.9 to 19.4)	0.078

a Adjusted for gender, age.

b Odds ratio can be interpreted as the odds of having quit smoking at the respective timepoint for the KISMET intervention compared to TAU.

### Secondary outcomes

#### Psychological outcomes

The KISMET intervention and TAU group did not differ significantly after 6 and 12 months for self-reported depression and anxiety (HADS), patient activation measure (PAM-13), and quality of life (SF-12) (Table 4). No significant differences were found at any time point for psychotic symptoms (PANSS-6). However, there was a statistically significant overall average difference for the PANSS-6. Throughout the study, participants in the KISMET group scored on average 2.9 (95% CI, 0.8 to 5.1) points higher than TAU participants.

**Table 4.** Adjusted mixed models^a^ at 6 and 12 months compared to baseline and average difference over time

Outcome	β (95% CI)		
	Baseline to 6 months	Baseline to 12 months	Average difference over time^b^
HADS depression subscale^c^	−0.1 (−1.6 to 1.4)	−0.8 (−2.3 to 0.8)	0.3 (−1.3 to 1.9)
HADS anxiety subscale^c^	0.9 (−0.7 to 2.4)	0.4 (−1.2 to 2.1)	1.3 (−0.5 to 2.9)
Psychotic symptoms^d^	−0.2 (−1.5 to 1.0)	1.0 (−0.3 to 2.4)	2.9 (0.8 to 5.1)
SF–12 mental component^e^	0.1 (−4.9 to 5.1)	−2.0 (−7.2 to 3.2)	−4.3 (−8.0 to −0.6)
SF–12 physical component^e^	1.1 (−2.5 to 4.7)	0.2 (−3.6 to 3.9)	−0.8 (−4.5 to 2.9)
Patient activation measure^f^	−0. (−5.1 to 4.5)	4.9 (−0.1 to 9.9)	0.6 (−3.9 to 5.0)
Systolic blood pressure	0.7 (−5.3 to 6.7)	−3.8 (−10.1 to 2.6)	−3.3 (−9.5 to 2.9)
Diastolic blood pressure	0.9 (−2.8 to 4.7)	−1.9 (−5.8 to 2.1)	1.3 (−2.5 to 5.0)
Body mass index	0.03 (−0.9 to 1.0)	−0.6 (−1.6 to 0.5)	1.1 (−1.5 to 3.6)
Fitness^g^	NA	3.5 (−26.0 to 32.9)	NA
Fasting glucose	NA	−0.3 (−0.8 to 0.3)	NA
Total cholesterol	NA	−0.2 (−0.9 to 0.5)	NA
HDL cholesterol	NA	0.1 (−0.2 to 0.3)	NA
LDL cholesterol	NA	0.3 (−0.4 to 0.9)	NA
Triglycerides	NA	0.2 (−0.9 to 1.4)	NA

a Adjusted mixed models corrected for gender and age.

b Unadjusted mean difference over time during 12-month study period.

c Scores range from 0 to 21, with higher scores indicating higher levels of anxiety or depression.

d Scores range from 6 to 42, with higher scores indicating more severe psychotic symptoms.

e Scores range from 0 to 100, with higher scores indicating better perceived quality of life regarding mental or physical health.

f Fitness assessed with the 6-minute walking test; distance covered in 6 minutes is presented in meters.

g Scores range from 0 to 100, with higher scores indicating better disease self-management.

#### Physiological outcomes

There were no statistically significant differences between the intervention and TAU group regarding systolic and diastolic blood pressure, body mass index, fitness, fasting glucose level, and lipid profile at any time point (Table 4).

### Attendance and smoking quit rates

Overall, participants attended a median of 9 (IQR = 10) out of 20 sessions delivered (minimum 0; maximum 20). A total of 35 participants (39%) had an attendance rate in the lowest tertile (0–6 sessions), 36 participants (40%) in the middle tertile (7–14 sessions), and 18 participants (20%) had an attendance rate in the highest tertile (15–20 sessions). All participants who quit smoking during the study period fell into the middle or highest tertile (Supplementary Materials S6).

### Quit attempts and pharmacological support

At 12 months, a total of 32 participants (86.5%) in the KISMET intervention reported to have made quit attempts, averagely 2.3 (SD 2.6) times in the past year, ranging between 0 and 12 quit attempts; 22 participants (73.3%) in the TAU group made quit attempts, 1.2 (SD 1.1) on average, ranging between 0 and 4 quit attempts.

At 12 months, 13 participants (35%) in the KISMET group reported the use of NRT (patches and chewing gum), six participants (16%) used Bupropion or Cytisine, and two participants (5%) combined NRT and Bupropion or Cytisine. In the TAU group 10 participants (33.3%) reported that they have used NRT (patches and chewing gum) (Supplementary Materials S7).

### Serious adverse events

Fifteen SAEs were recorded (nine in the intervention and six in the TAU group), out of which four events were classified as serious, one as life-threatening, and one as fatal. However, all except one of these events were assessed to be unrelated to the study (Supplementary Materials S8). Two patients were admitted to a psychiatric ward because of a psychotic episode. One was admitted because of a deterioration in somatic health; one patient died from alcohol intoxication. One adverse event was considered by MHPs to be possibly related to the study, specifically to psycho-education on smoking and health risks. In this case, information about the potential risks of passive smoking caused a patient to feel excessive guilt and responsibility for a friend who had received an abnormal lung CT scan. Unfortunately, this patient with SMI subsequently experienced a psychotic episode.

### Dropouts

In the intervention group, 52 participants (58%) had dropped out throughout the trial, with the majority dropping out within the first 3 months (*n* = 36). In the TAU condition, a total of 14 participants (32%) had dropped out. Worsening mental health problems, a serious adverse event, or psychiatric hospitalization accounted for 24% of all participants who dropped out in both conditions. Other reasons included a loss of motivation (15%) and no confidence in their capacity to quit smoking (10%). In particular, four patients dropped out from the KISMET intervention because of having doubts about the group setting, or because they were dissatisfied with the organization of the group by MHPs. Fifteen participants (23%), who had dropped out could not be reached.

## Discussion

The aim of this study was to determine the effectiveness of the KISMET smoking cessation intervention compared to treatment as usual in mental healthcare teams. The intervention group showed higher quit rates than the TAU group throughout the 1-year study period, with significant differences at three and 12 months follow-up but not at 6 months. No statistically significant or clinically relevant differences between the groups were found for any of the secondary outcomes. These findings highlight two important messages: (1) the KISMET intervention seems to be superior to TAU for smoking cessation support in FACT teams and (2) the KISMET intervention did not have detrimental effects on patients’ mental health.

Our results contribute to the evidence that people with SMI can achieve long-term smoking cessation despite the great effort that is needed to achieve this, both on a patient level with up to 12 quit attempts, and on a treatment level with high dropout (Daumit et al., 2023; Gilbody et al., 2019; Spanakis et al., 2022). The treatment effect was most pronounced at 3 months, which may reflect the intensive intervention phase during this period, characterized by weekly sessions. Treatment effects were more pronounced at 12 months than at 6 months, indicating that smoking cessation can be a slow process that needs time. It may also indicate a remaining selection of participants who are abstinent at 12 months, thereby potentially inflating the quit rate. Many participants dropped out because they gave up on attempting to quit smoking and therefore did not wish to continue in the study. Even so, when participants who dropped out were treated as currently smoking in the analysis, effect sizes were only slightly attenuated or remained similar. Our findings differ from the SCIMITAR+ trial, which reported higher odds ratio at 6 months, and demonstrated a fading in treatment effect over time with lower odds ratio at 12 months (Gilbody et al., 2019, 2021).

In line with recent studies, we did not find differences in mental health outcomes between the allocation groups at any time point, including depression, anxiety, and psychotic symptoms (Gilbody et al., 2019; Taylor et al., 2014). Our findings therefore support that smoking cessation interventions do not have detrimental effects on mental health countering concerns that smoking cessation might worsen mental health (Peckham et al., 2019; Taylor et al., 2021; Wu, Gao, Aveyard, & Taylor, 2023). We also found no relevant differences in quality of life, disease self-management or physical health outcomes, including blood pressure and lipid profile, similar to the SCIMITAR+ trial (Gilbody et al., 2019). As in the SCIMITAR+ trial, engagement and uptake of available smoking cessation support in our study, such as the prescription of smoking cessation medication, were considerably greater in the intervention group than in the TAU group, endorsing the systematic and proactive delivery (Peckham et al., 2019). This trial has a number of strengths. First, our study was a pragmatic trial conducted under real-world circumstances regarding human and financial resources, thereby increasing its generalizability to similar mental healthcare settings. Second, we collected data on depression, anxiety, and psychotic symptoms, providing comprehensive insights into potential differences and changes in mental health, which is of high importance in this setting (Taylor et al., 2021). Third, the relatively comparable baseline characteristics of participants in the KISMET and TAU group indicate that recruitment bias is unlikely (Puffer, Torgerson, & Watson, 2003), although there is a difference in distribution in diagnoses with 39% of participants having a schizophrenia diagnosis in the TAU group and 59.2% in the KISMET group. In addition, participants in the experimental group had slightly more severe psychotic symptoms than participants in the control group at baseline (see Table 1). This indicates a slight overrepresentation of people with less severe symptoms in the TAU group. Finally, we used CO monitoring to biochemically verify self-reported smoking status, which additionally turned out to be a valuable tool for MHPs and patients. The usefulness of (self-)monitoring has been demonstrated in other studies involving smoking and other substance use (Beard & West, 2012; Gass et al., 2021).

Our study also has some limitations. The first limitation is the sample size (*N* = 133), which turned out smaller than initially planned (Küçükaksu et al., 2023). We also experienced considerable loss to follow-up. Dropout of 49% (*N* = 66), of which most in the intervention group (*N* = 52), was much higher than expected and, although slightly more, still comparable to studies conducted in similar settings (Gilbody et al., 2015, 2019).

In the context of a process evaluation, we investigated the underlying reasons for the sparse inclusion and high dropout rate. FACT teams reported systemic hardship due to shortage of staff, high employee turnover rates and the re-structuring of mental healthcare organizations due to a new reimbursement system (‘Zorgprestatiemodel: de bekostiging voor ggz en fz’, 2022), which negatively impacted the available human resources and time for patient recruitment (Küçükaksu et al., 2025b). Additionally, MHPs reported that many patients did not want to commit to a smoking cessation group and/or in the context of a research project, even if they had the wish to quit. Reasons why patients declined to participate in the study were not systematically recorded. Reasons for dropping out of the study included an exacerbation of mental health problems (unrelated to the intervention), loss of motivation to quit, and lack of confidence in one’s capacity to quit. The high dropout rate specifically in the intervention group may also indicate that, for some patients, the burden of the intervention was too high and that perhaps motivational interviewing skills of MHPs should be improved (Küçükaksu et al., 2025b). Difficulties in recruitment and loss to follow-up, are also indicative of underlying potential dilemmas to commit and adhere to an intervention while still being addicted (to nicotine). This illustrates an ambivalence about and fluctuations in motivation to quit smoking that are inherent to addiction (Veilleux & Steggerda, 2023; Vermeulen, 2024). To illustrate, a pragmatic trial on a lifestyle intervention focused on weight loss in the same study population and setting, the SMILE study, was able to recruit more patients within the same number of participating FACT teams and had an overall loss to follow-up of 24% (Walburg et al., 2023). Another limitation is the absence of an active control group for comparison with the KISMET intervention, which may have amplified the observed treatment effects. In a review, bespoke face-to-face interventions for people with SMI were superior compared to TAU conditions, but no conclusive evidence was found when compared to other interventions (Spanakis et al., 2022). Nevertheless, the aim of this trial was to examine the added value of the KISMET intervention over the current standard practice in FACT teams, making TAU the most appropriate control condition (Cuijpers et al., 2021). Finally, we did not collect data on the doses of transdermal nicotine patches. The treating psychiatrist or clinical nurse specialist determined the dose according to the number of cigarettes per day currently smoked, in line with medical guidelines. A schedule was made together with the patient to gradually decrease the dose. There is evidence, however, that higher dosages of nicotine patches are more effective and that a combination of fast-acting NRT and nicotine patches are more effective than single form of either (Theodoulou et al., 2023). This should be taken into account to maximize the potential effectiveness of NRT.

The component of peer support was carried out under suboptimal circumstances due to limited availability of experts-by-experience in many FACT teams. Nevertheless, it has been received positively by patients and professionals further advocating the integration of peer support for smoking cessation. Group-based social support can contribute to successful smoking cessation (Küçükaksu et al., 2025a; Leutwyler et al., 2025). The three intervention components, including implications for practice and experiences of patients and professionals with the intervention, are discussed in more depth in our process evaluation (Küçükaksu et al., 2025a, 2025b).

There is research that suggests that some people with SMI might benefit from a flexible approach to smoking cessation focusing on initial smoking reduction, as a first step toward cessation (McChargue, Gulliver, & Hitsman, 2002; Tidey & Miller, 2015). Although gradual smoking cessation was encouraged within the KISMET intervention, the goal was full smoking cessation. Future interventions could additionally focus on smoking reduction, thereby increasing the willingness of patients to continue with the intervention, even though they are not ready yet to quit smoking altogether. To ensure bespoke smoking cessation support, mental healthcare providers should encourage those specific patients who feel ambivalent about quitting smoking to set goals focused on smoking reduction while endeavoring smoking cessation as a long-term goal.

### Implications for research

Most importantly, future research should invest more in improving recruitment and retention strategies. These may include additional motivational enhancement for patients, and the promotion of a flexible approach to smoking cessation. For successful recruitment, positive attitudes of MHPs toward smoking cessation, more application of motivational strategies, and enhancing a strong relationship between MHPs, researchers and patients are essential (Küçükaksu et al., 2025b; Peckham et al., 2018). Furthermore, future studies should consider extended follow-up periods (e.g. 18 and 24 months) to detect improvements in mental and physiological parameters (Daumit et al., 2023).

Overall, our results show that the KISMET intervention has a more favourable effect on smoking cessation after 12 months compared to TAU. To draw more solid conclusions about its effectiveness, enhancing participant recruitment and retention is crucial.

## Supporting information

10.1017/S0033291726104516.sm001 Küçükaksu et al. supplementary material Küçükaksu et al. supplementary material

## Data Availability

Data supporting this study can be made available by the corresponding author.

## Supplementary material

The supplementary material for this article can be found at http://doi.org/10.1017/S0033291726104516.
