# Supplementary material for: Effectiveness of a one-year smoking cessation intervention for people with severe mental illness: results of the KISMET cluster-randomized controlled trial
Source: Psychol Med. 2026 May 15;56:e158. doi: 10.1017/S0033291726104516 (PMC13200142; doi:10.1017/S0033291726104516)
Supplement: Küçükaksu et al. supplementary material [file S0033291726104516sup001.docx]

**Supplementary material**

**S1.** Overview KISMET intervention content

**S2.** Total characteristics of Complete Cases and Dropouts

**S3.** Characteristics of Complete Cases and Dropout in the intervention group

**S4.** Characteristics of Complete Cases and Dropout in the TAU group

**S5.** Smoking quit rates at three, six and 12 months in both groups

**S6.** Attendance at group sessions and smoking quit rates

**S7.** Use of pharmacological support

**S8.** Serious adverse events (SAEs) and relation to study

These supplementary materials have been provided by the authors to give additional information about the data this work is based on.

**S1.** **Overview KISMET intervention content**

| **Weekly sessions (initial phase)** | **Content of sessions** |
| --- | --- |
| Session 1 | Introduction and personal motivation |
| Session 2 | Psycho-education and pharmacological treatment for smoking cessation (part I) |
| Session 3 | Psycho-education and pharmacological treatment for smoking cessation (part II) |
| Session 4 | Action plan and practical tips for a quit attempt |
| Session 5 | Relapse prevention and coping strategies |
| Session 6 | Pros and cons of (not) smoking |
| Session 7 | Smoking and mental health |
| Session 8 | Recap of motivation and relapse prevention strategies |
| Session 9 | The function of smoking in emotion regulation |
| Session 10 | Smoking cessation and a healthy lifestyle |
| Session 11 | Smoking in relation with other substance use |
| Session 12 | Self-esteem and confidence |
| Session 13 | Reflection, CO monitoring and future goals |
| **Monthly sessions**  **(maintenance phase)** |  |
| Session 14 – 20 | Recap of earlier sessions and new input based on patients’ experiences |

**S2.** Baseline characteristics of complete cases and dropouts (total)

| **Variable** | **Complete** | **N**  **(total= 67)** | **Dropouts** | **N**  **(total= 66)** |
| --- | --- | --- | --- | --- |
| **Age, mean (SD)** | 48.1 (11.1) | 66 | 45.3 (12.1) | 49 |
| **Gender, male, n (%)** | 40 (40.3) | 67 | 22 (44.9) | 49 |
| **Gender, female, n (%)** | 27 (59.7) | 67 | 27 (55.1) | 49 |
| **Body Mass Index, mean (SD)** | 28.7 (5.9) | 65 | 28.4 (7.8) | 46 |
| **Average number of cigarettes per day, mean (SD)** | 18.5 (9.7) | 65 | 18.2 (11.1) | 48 |
| **FTND, mean (SD)** | 5.8 (2) | 65 | 5.9 (1.7) | 47 |
| **Years smoking** | 28.1 (11.8) | 65 | 24.8 (12.7) | 48 |
| **Depression (HADS), mean (SD)** | 6.7 (4.5) | 66 | 8.5 (4.7) | 48 |
| **Anxiety (HADS), mean (SD)** | 8.3 (5.1) | 66 | 9.5 (5.1) | 48 |
| **Psychotic symptoms (PANSS), mean (SD)** | 11.6 (5.3) | 61 | 13.3 (7.1) | 43 |

Complete case: participants with data available at 12-months follow-up
Dropout: participants without data available at 12-months follow-up

**S3.** Baseline characteristics of complete cases and dropouts in the intervention group

| **Variable** | **Complete** | **N**  **(total= 37)** | **Dropouts** | **N**  **(total N= 52)** |
| --- | --- | --- | --- | --- |
| **Age, mean (SD)** | 47.1 (11.4) | 37 | 47.4 (11.6) | 37 |
| **Gender, male, n (%)** | 19 (51.4) | 37 | 16 (43.2) | 37 |
| **Gender, female, n (%)** | 18 (48.6) | 37 | 21 (56.8) | 37 |
| **Body Mass Index, mean (SD)** | 28.6 (6.7) | 35 | 29.1 (8.2) | 35 |
| **Average number of cigarettes per day, mean (SD)** | 19 (9.5) | 35 | 20 (11.8) | 37 |
| **FTND, mean (SD)** | 6 (2) | 36 | 6.3 (1.6) | 35 |
| **Years smoking** | 26.2 (10.3) | 36 | 26.8 (12.5) | 36 |
| **Depression (HADS), mean (SD)** | 6.4 (4) | 36 | 9 (4.6) | 37 |
| **Anxiety (HADS), mean (SD)** | 8.7 (4.8) | 36 | 10 ( 5) | 37 |
| **Psychotic symptoms (PANSS), mean (SD)** | 12.3 (5.1) | 31 | 14.2 (7.7) | 33 |

Complete case: participants with data available at 12-months follow-up
Dropout: participants without data available at 12-months follow-up

**S4.** Baseline characteristics of complete cases and dropouts in the TAU group

Complete case: participants with data available at 12-months follow-up
Dropout: participants without data available at 12-months follow-up

| **Variable** | **Complete** | **N**  **(total N= 30)** | **Dropouts** | **N**  **(total N=14)** |
| --- | --- | --- | --- | --- |
| **Age, mean (SD)** | 49.4 (10.8) | 29 | 38.8 (12.1) | 12 |
| **Gender, male, n (%)** | 21 (70) | 30 | 6 (50) | 12 |
| **Gender, female, n (%)** | 9 (30) | 30 | 6 (50) | 12 |
| **Body Mass Index, mean (SD)** | 28.7 (4.9) | 30 | 26 (6.1) | 11 |
| **Average number of cigarettes per day, mean (SD)** | 18 (10) | 30 | 14 (7.2) | 11 |
| **FTND, mean (SD)** | 5.5 (2) | 29 | 4.8 (1.6) | 12 |
| **Years smoking** | 30.6 (13.2) | 29 | 19 (12.2) | 12 |
| **Depression (HADS), mean (SD)** | 7.1 (5.2) | 30 | 7.6 (4.9) | 11 |
| **Anxiety (HADS), mean (SD)** | 7.7 (5.4) | 30 | 9.6 (5.6) | 11 |
| **Psychotic symptoms (PANSS), mean (SD)** | 10.9 (5.4) | 30 | 10.4 (3.8) | 10 |

**S5.** Smoking cessation prevalence (%) at 3-, 6- and 12-months’ follow-up

**S6.** Attendance at group sessions and smoking quit rates at 3-, 6- and 12-months’ follow-up

Lowest tertile: 0-6 sessions
Middle tertile: 7-14 sessions
Highest tertile: 15-20 sessions

**S7.** Use of smoking cessation medication at 12 months

**S8.** Serious adverse events (SAEs) and relation to study

|  | **KISMET**  **(n=74)** | **TAU**  **(n=42)** | **Total** |
| --- | --- | --- | --- |
| Serious adverse events |  |  |  |
| Definitely related | 0 | 0 | 0 |
| Possibly related | 1 | 0 | 1 |
| Unrelated | 6 | 6 | 12 |
| Unclassified | 2 | 0 | 2 |
| Total | 9 | 6 | 15 |
